# Measurement of Lead Localization Accuracy Based on Magnetic Resonance Imaging

**DOI:** 10.3389/fnins.2021.632822

**Published:** 2021-12-22

**Authors:** Changgeng He, Feng Zhang, Linze Li, Changqing Jiang, Luming Li

**Affiliations:** ^1^National Engineering Laboratory for Neuromodulation, School of Aerospace Engineering, Tsinghua University, Beijing, China; ^2^Precision Medicine and Healthcare Research Center, Tsinghua-Berkeley Shenzhen Institute, Tsinghua University, Shenzhen, China; ^3^IDG/McGovern Institute for Brain Research at Tsinghua University, Beijing, China; ^4^Institute of Epilepsy, Beijing Institute for Brain Disorders, Beijing, China

**Keywords:** deep brain stimulation, magnetic resonance imaging, lead localization, artifact, template

## Abstract

Post-implantation localization of deep brain stimulation (DBS) lead based on a magnetic resonance (MR) image is widely used. Existing localization methods use artifact center method or template registration method, which may lead to a considerable deviation of > 2 mm, and result in severe side effects or even surgical failure. Accurate measurement of lead position can instantly inform surgeons of the imprecise implantation. This study aimed to identify the influencing factors in DBS lead post-implantation localization approach, analyze their influence, and describe a localization approach that uses the individual template method to reduce the deviation. We verified that reconstructing direction should be parallel or perpendicular to lead direction, instead of the magnetic field. Besides, we used simplified relationship between magnetic field angle and deviation error to correct the localization results. The mean localization error can be reduced after correction and favors the feasibility of direct localization of DBS lead using MR images. We also discussed influence of *in vivo* noise on localization frequency and the possibility of using only MR images to localize the contacts.

## Introduction

Deep brain stimulation (DBS) is a widely used treatment for various neurological and neuropsychiatric diseases, including Parkinson’s disease, epilepsy, and depression (Benabid, 2003; Stefurak et al., 2003; Huys et al., 2016). Its clinical outcomes depend on the regulation of disease-specific pathological neural circuits through precise stimulation of the artfully selected targets (Knight et al., 2015). Accurate implantation of stimulating leads is required in order to have a favorable clinical outcome. In addition, the spatial relationship between the leads and target nuclei, as well as the surrounding neural elements, should also be determined to guide the programming in order to optimize clinical efficacy and reduce side effects (Butson et al., 2007; Chaturvedi et al., 2010). It can also help elucidate the therapeutic mechanism of DBS and its related fundamental brain functions.

Postoperative magnetic resonance imaging (MRI) is generally used to localize implanted leads (Pollo et al., 2004; Starr et al., 2010). It could provide simultaneous and direct visualization of the leads and the surrounding brain tissues. In addition, results can be readily fused with those from other MRI modalities, such as functional MRI, in order to provide powerful means of investigating the DBS and disease mechanisms (Schönecker et al., 2014; Ashkan et al., 2017). Safety concerns once hindered its use in clinics, which led to the proposal of alternative approaches, such as fusion of computed tomography (CT) and preoperative MRI techniques (Thani et al., 2011; Engelhardt et al., 2019). Nowadays, after continuous efforts to address these concerns and with the new designs of the DBS device (Jiang et al., 2013; Zhao et al., 2020), not only 1.5 T but also 3.0 T MRI is now deemed relatively safe under certain controlled conditions (Jiang et al., 2014; Mo et al., 2016). An increasing number of clinical centers are performing MRI after DBS implantation as a routine procedure (Lozano et al., 2019).

With less obvious safety concerns, accuracy of direct lead localization on MR images becomes the major question. The metallic contacts of the lead can induce large artifacts on the images due to the magnetic susceptibility difference between the contacts and surrounding brain tissues (Schenck, 1996), which would hinder the identification of the actual contact positions. Generally, the contact artifacts are treated as ellipses symmetrical to the lead axis and whose centers are deemed coincidence with those of the contacts (Pollo et al., 2004). However, this is not the exact case when the lead is inclined from the main field direction of the MRI. Not only are the shapes of the artifacts rather irregular but also the centers would deviate from the true positions of the contacts (Matsui et al., 2007; Lee et al., 2010). Moreover, a number of factors, such as reconstruction direction, sequence type, and scan parameters, would also affect the appearance of the artifact, making it even challenging to address the problem (Liu et al., 2001).

The average template method is widely used in postoperative MR localization (Horn and Kühn, 2015). However, this method may ignore some details of the electrode artifacts. Therefore, the registration result may not be as good as the personalized template.

In order to evaluate the accuracy of direct lead localization on postoperative MR images, phantom experiments were conducted under 3.0 T MRI in this study. The DBS lead was placed at a variety of orientations, and both T1- and T2-weighted images were acquired. Positions of the contacts directly derived from the artifacts were compared with those calculated based on fiducial marks, which were more accurate measurements, to get the localization error. Our design guaranteed that the calculation of contact center coordinate is accurate and provide a reliable reference to estimate the localization error in the traditional method, which was not considered in previous studies. Through these analyses, whether and how MR images alone can be used as a reliable lead localization method was discussed.

## Materials and Methods

### Deep Brain Stimulation Lead

The lead PINS L301 (PINS Medical, Inc., Beijing, China) was used in the experiments. Four cylindrical-stimulating contacts (distal contact 0 to proximal contact 3) made of platinum–iridium alloy was established in the distal end. Each contact was 1.5 mm in length and 1.27 mm in diameter. The space between the two adjacent contacts was 0.5 mm. The proximal end of the lead bore the four connecting contacts. Both ends were interconnected by helical platinum–iridium alloy cables covered with polyurethane sheath.

### Experiment Setup

A cuboid-shaped phantom with inner dimensions of 280 × 170 × 150 mm^3^ was made and filled with a solution consisting of 5 g/L of CuSO4 and 0.9 g/L of NaCl. It was placed flat with the long axis parallel to the main field B0 and calibrated with a spirit level.

**FIGURE 1:**
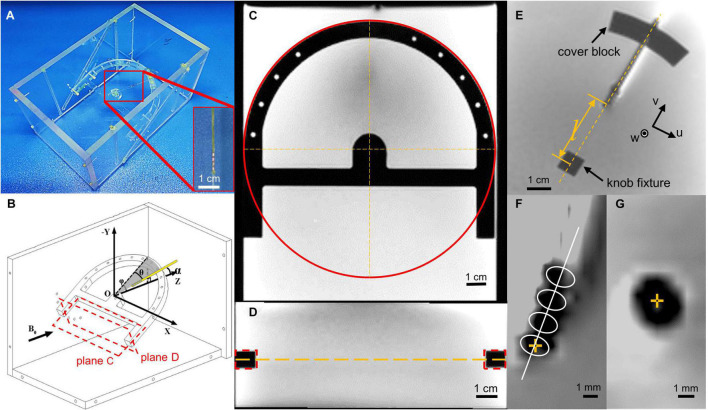
Illustration of the experiment layout. **(A)** Photo of the phantom layout with the lead terminal tied to the nylon wire and fixed on a cubic fixture. **(B)** The schematic map of the experiment layout. Black vector stands for the direction of the main field. Plane **(C,D)** stands for the cross sections in **(C,D)**. **(C)** Demonstration of how we calculate the fixture coordinate system center in a slice and axis direction. The red circle stands for the Hough transformation, which gives us the coordinate of the fixture center. **(D)** Demonstration of how we calculate the slice that contains the coordinate system center. There d-dotted rectangle stands for the cross section of the fixture, which gives the center slice position, which contains the lead. **(E)** Magnetic resonance (MR) image of the lead, cover block, and knob fixture, and depiction of the u and v axes, whereas the w-axis is perpendicular to the u–v plane. Length l indicates the distance between contact 0 and the center of the knob fixture, measured using an image measuring instrument. **(F)** The template used to directly identify the contact center in the coronal view. The center of contact 0 is highlighted with a yellow cross mark. **(G)** The contact center is in transverse view, highlighted with a yellow cross.

To hold the lead at various orientations, a rotatable fixture was designed as presented in Figures 1A,B. The lead was clamped on the ring of the rotatable fixture with a cover block. A nylon wire was tied to the tip of the lead and fixed to a knob fixture at the center of the arc in order to maintain the lead straight. A coordinate system could be established with the origin located at the center of the knob fixture and Z-axis aligning with B0. By adjusting the cover block position and the knob fixture orientation, the lead could be deflected to an angle θ of 0°, 15°, and 30° relative to the Y–Z plane. Its inclination angle φ relative to the horizontal plane could be adjusted to 0°, 15°, 30°, and 45° by fixation to location holes on the sidewalls with nylon screws. The angle α of the lead axis relative to the B0 direction can be calculated by


(1) $$\alpha =\cos^{-1}\left(\cos\varphi \,\cos\theta \right)$$

The phantom and fixtures were made of PMMA, which had little artifact in MR images, so that the profiles could serve as fiducials to determine the lead positions. Before scanning, air bubbles were removed to prevent their influence on images.

### Magnetic Resonance Data Acquisition

T1-weighted three-dimensional turbo field echo (T1w-3D-TFE) and T2-weighted turbo spin echo (T2w-TSE) sequences were scanned on a 3.0 T MRI scanner (Philips Ingenia, Netherlands, software version 6.0.541.1), with a 32-channel head coil (NMRB 375, Philips, Netherlands). The parameters for the T1w-3D-TFE sequence were repetition time (TR) of 10.2 ms, echo time (TE) of 4.7 ms, field of view (FOV) of 224 mm, matrix of 224 × 224, and slice thickness of 1 mm, resulting in a final voxel size of 1 × 1 × 1 mm^3^. The parameters for the T2w-TSE sequence were TR of 2,111 ms, TE of 90 ms, FOV of 280 mm, matrix of 720 × 720, and slice thickness of 2 mm, resulting in a final voxel size of 0.39 × 0.39 × 2 mm^3^.

### Contact Localization

The center of contact 0 was localized longitudinally and transversely from T1 and T2 images, respectively. All reconstructed images were bilinear, upsampled to a resolution of 0.1 mm before processing.

In order to locate the contacts in the image, we created a fixture coordinate system. We used multiple cross-section images to establish the rotatable fixture coordinate system. The cross section parallel to the rotatable fixture was used to determine the axis of the knob fixture in the image slice. We used the Hough transform to calculate the center of the half-ring structure and the bar direction of the rotatable fixture (Figure 1C). The cross-section perpendicular to the rotatable fixture and parallel to the x-axis was used to calculate the slice, which contains the lead (Figure 1D). The center of the knob fixture was selected as our origin of the fixture coordinate system, and the axis is determined by the bar direction and fixture symmetry axis. After the coordinate system is set, we calculate the coordinates of the contact with parameters measured with a microscope. As with the fiducial approach, the longitudinal position of contact 0 was determined by offsetting the center of the knob fixture O along the lead axis by the distance l between the center of contact 0 and O, which was precisely premeasured when the lead was fixed on the fixtures using an image measurement instrument (Optiv Advance 332, Hexagon AB, Sweden). For direct identification, a template-based approach was used. A template consisting of a series of four identical ellipses equally spaced by 2.0 mm with their axes perpendicular to the lead axis was used, as presented in Figure 1D. The position of contact 0 with the highest correlation between the projections of the artifact and the template was searched along the lead axis. The longitudinal position deviation of contact 0 between the two methods was then calculated and denoted by dL.

On the transverse T2 images, the position of contact 0 was either fiducially determined from the intersection of the lead axis and the image plane, or directly identified by picking the center of the hypointense artifact as presented in Figures 1E–G. The deviation between the two methods was denoted by dT.

We computed contrast-to-noise ratio (CNR) and analyzed the influence of CNR on localization error. Coronal view images were selected to study this influence since they were frequently used in localization. We calculated CNR in both phantom and *in vivo* conditions, and simulated *in vivo* condition by adding artificial noise to the phantom data. The noise is sampled from location where there is no contrast media, where the signal should be zero, and all signals can be considered noise (National Electrical Manufacturers Association, 2008). After calculating the standard deviation from the noise, we derived CNR from both phantom and *in vivo* data. Artificial Gaussian noise was added to the phantom data so that they have the same CNR as the *in vivo* data. Then we compared the localization error between the phantom data and simulated *in vivo* data.

### Template Data Processing

#### Deviation From the Real Position

The lead was registered to a template using the imregister function in MATLAB (MathWorks, Natick, MA, United States) software. The center of contact after registration was chosen as the focus. The deviation was calculated for the registration results of both the mean template and individual template registration methods. We selected an individual template by choosing the closest direction in our experiment setup, and the mean template is the average of all 12 templates generated in the phantom experiment. The deviation is defined by the difference between the real position and registration calculated position. The real position was calculated using the lead fixture as reference. The registration calculated position was derived by the near template registration algorithm.

#### Correlation Coefficient Calculation

We compute 12 × 12 pairwise correlation coefficient matrixes for direction correlation, template volume correlation, and template volume correlation after registration in order to measure their similarity.

### Statistical Analysis of the Phantom Experiment

Coordinate values were expressed as mean ± standard deviation (SD). Two-way analysis of variance (ANOVA) was conducted to investigate the influence of the θ and φ angles on the localization deviation, including their interaction effect. The statistical significance level was set at 0.05. All calculations were performed using the MATLAB software (MathWorks, Natick, MA, United States).

## Results

### Longitudinal Localization of the Lead Contact

**FIGURE 2:**
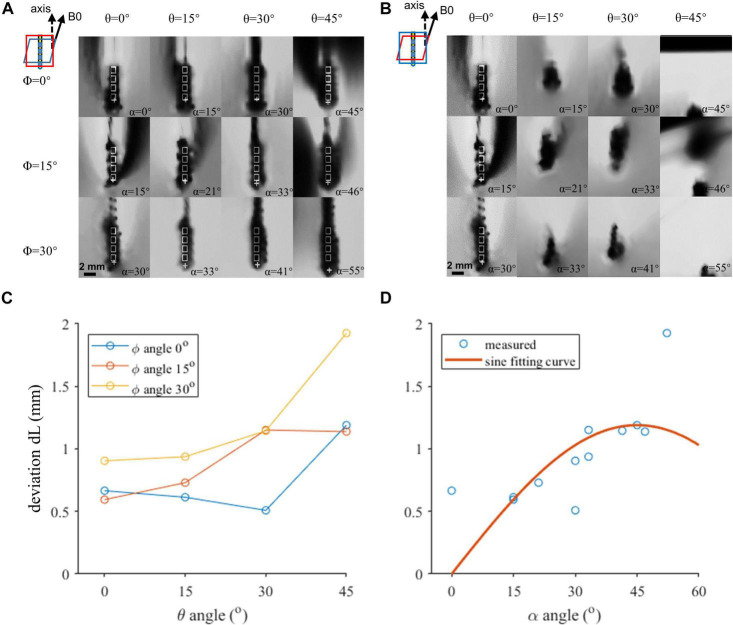
The coronal view of the artifacts of four contacts in various orientations. **(A,B)** Artifact positions under different lead orientations. White rectangles indicate the outline of the contacts. Cross markers indicate the template-matched position of the proximal contact. **(A)** Image resliced parallel to the top surface of the rotatable fixture. **(B)** Image resliced parallel to the X–Z plane. **(C,D)** Localization deviation as a function of the axis direction. **(C)** Horizontal axis indicates the θ angle. Three curves represent different φ angles. Both θ and φ angles contribute to the deviation, and reduction of either of the angles can achieve a better accuracy. **(D)** Horizontal axis indicates α. The red plane in the zoomed-in figure shows the reconstruction plane we chose in each condition in **(A,B)**.

The regions of the T1w-3D-TFE images containing the lead contacts at various lead orientations are shown in Figures 2A,B, which were reconstructed along the lead axis and along the B0 direction, respectively. The hypointense artifacts of the four contacts could be clearly seen in Figure 2A, but were hard to distinguish from Figure 2B. It indicated the importance of the reconstruction direction on the accuracy of lead localization, and contact positions were determined from Figure 2A by both the fiducial-based and direct identification approaches, and marked in the figure. The directly identified positions of contact 0 deviated from the fiducial results by 0.51 to 1.93 mm at various orientations, as shown in Figure 2D. Two-way ANOVA revealed that both θ and φ had significant influence on dL (*p* = 0.003 and 0.017, respectively). It could further be seen from Figure 2C that dL showed an increase with the angle α, which combined θ and φ according to Equation (1).

### Transverse Localization of the Lead Contacts

**FIGURE 3:**
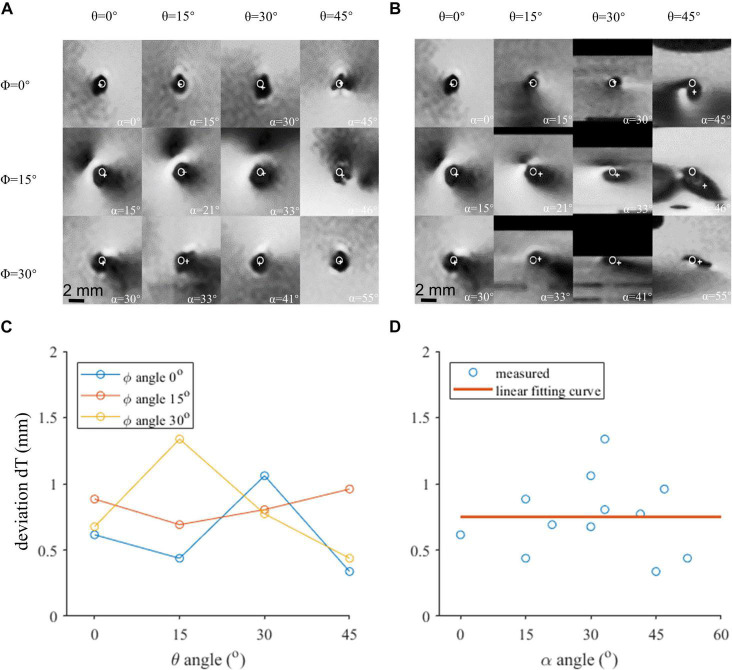
The axial view of the contact 0 artifact in various orientations. **(A)** Image reconstructed perpendicular to the lead axis. **(B)** Image reconstructed perpendicular to the B0 field. **(C)** Localization deviation assessed in the axial view under different θ and φ angles. **(D)** The localization deviation of contact 0 with respect to α, reconstructed perpendicular to the B0 field.

The regions of the T2w-TSE images containing the lead contacts are shown in Figures 3A,B, which were reconstructed perpendicular to the lead axis and the B0 direction, respectively. The profiles of the artifact were relatively regular in Figure 3A, but were severely distorted in many of the images in Figure 3B, especially in those with large α angles. Thus, transverse contact positions were determined from and marked in Figure 3A. There was a slight difference of 0.75 ± 0.29 mm between the directly identified and fiducial-derived positions, as shown in Figure 3D. Neither φ nor θ angle showed significant impact on dT, which was consistent with the influence of α, as shown in Figure 3C.

### Contrast-to-Noise Ratio Influence on Localization Error

**FIGURE 4:**
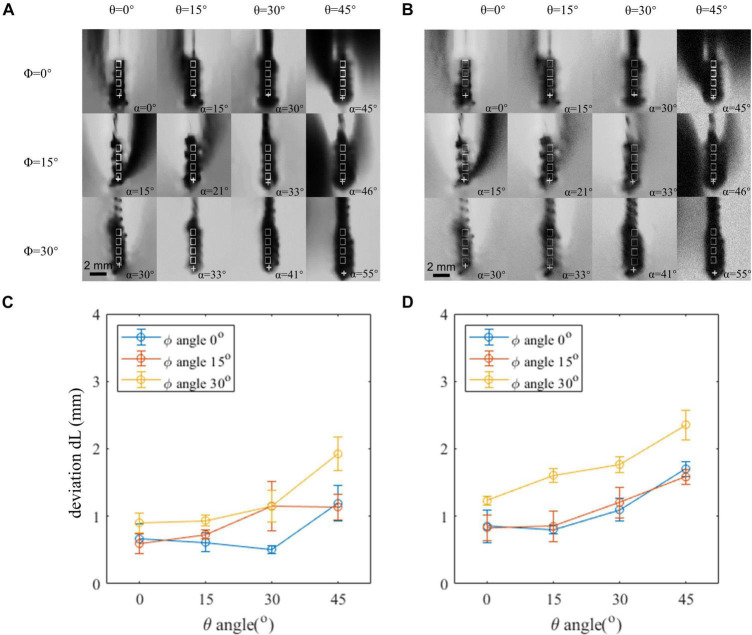
Comparison between the phantom image and *in vivo* contrast-to-noise ratio (CNR)-mimicked image. **(A)** Artifact positions under different lead orientations in the phantom image. **(B)** Artifact positions under different lead orientations in the phantom image, which added an artificial noise to mimic the *in vivo* condition. **(C,D)** Localization error of the original phantom image and phantom image-added artificial noise. Horizontal axis indicates the θ angle. Three curves represent different φ angles. Both θ and φ angles contribute to the deviation, and reduction of either of the angles can achieve a better accuracy.

We estimated the CNR and noticed that the CNR is different between the phantom data and clinical *in vivo* data. The CNR estimated is around 60 from the phantom data and 20 from the clinical data. We added Gaussian noise to the phantom data and decreased its CNR to 20 to mimic the condition of the *in vivo* data. The generated *in vivo*-like images are shown in Figure 4B. We compared the localization error between the phantom data and the mimicked *in vivo* data in Figure 4. The overall localization error increased after adding more noise. The largest localization error before and after correction are 2.4 and 1.6 mm, respectively. The mean error after correction increased from 0.42 to 0.79 mm.

### Comparison Between Different Templates

**FIGURE 5:**
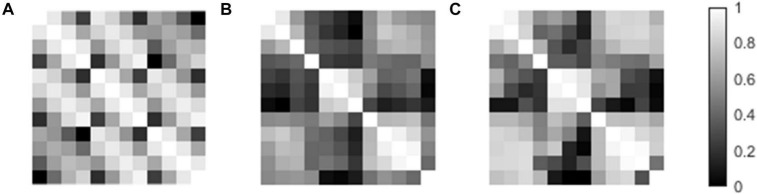
The template paired correlation coefficient matrixes. **(A)** Paired matrix of the template direction vector. **(B)** Paired matrix of the template volume. **(C)** Paired matrix of template volume after registration.

**FIGURE 6:**
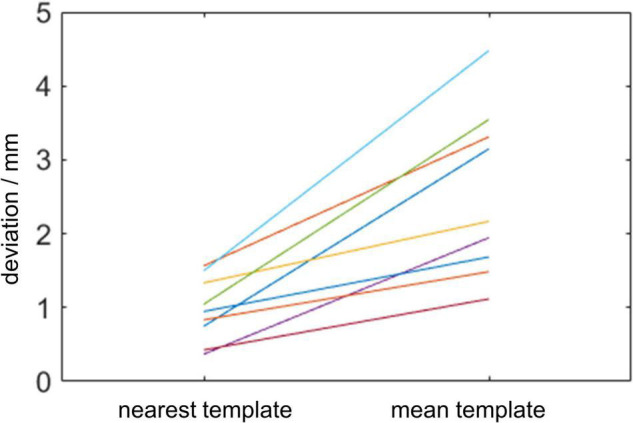
Deviation of the center of proximal contact before and after registration.

The 12 × 12 correlation matrix show pairwise correlation of all individual templates (Figure 5). All 12 templates were registered to each other, and correlation coefficients were calculated after registration. This similarity measurement provides us the explanation of the difference between mean template and near template. In image registration algorithm based on intensity, similarity can give us a hint on the potential registration behavior. In our condition, the localization error depends on registration accuracy, which can be reflected in template similarity. The individual template was used to mimic the widely used template matching method. However, the result is not as good as the individual template result. The best individual template registration result exceeds the result of the mean template registration. The mean template has lower correlation coefficient than the best correlated template (Figure 6).

## Discussion

Knowing the accurate location of the implanted DBS lead inside the target nucleus is important to understand the mechanisms and promote the therapy development. Direct localization from MR images is the handiest way but is hindered by electrode artifacts.

In this study, we designed phantom experiments with fiducial frames to guarantee direct localization accuracy of the DBS lead from MR images. Most previous studies focused on CT/MR image fusion localization error (Lee et al., 2010; Smith and Bakay, 2011), but our study intends to explore the possibility of localization with only MR images. Former studies used phantom experiments, which gives accurate reference for localization of lead but only in the parallel and perpendicular directions (Pollo et al., 2004, 2007). In our study, we took the typical lead direction range into consideration and analyzed the relationship between localization errors. Besides these analyses, we used simplified relationship to decrease the error to the level of 0.42 mm on average, which favors the feasibility of direct localization of the DBS lead from MR images. We also analyzed the localization error difference between the phantom study and *in vivo* condition by adding artificial noise to mimic the *in vivo* condition. The phantom experiment paradigm may also be used for further applications, such as generating artifact template for localization by registration method.

We conducted a systematic analysis on error propagation in the phantom experiment from two aspects: the accuracy of the phantom localization system and the reliability of the manually mimicking traditional localization method.

As for the phantom localization system, the propagation of error is from the phantom to fiducial points to the calculation of the contact center. The phantom and the lead fixture were machined with an accuracy of 0.1 mm, which is guaranteed by the machine tool. After upsampling the MR image to a resolution of 0.1 mm, the center of both the knob fixture and cover block have an accuracy of 0.1 mm in both u and v directions in Figure 1E. Thus, the uncertainty of lead position calculation in each direction can be derived by:


(2) $$\sqrt{k^{2}\,\sigma^{2}+{\left(1-k\right)}^{2}\,\sigma^{2}}=\sigma \,\sqrt{k^{2}+{\left(1-k\right)}^{2}}<\sigma ,0\leq k\leq 1$$

in which k is the proportion of length l in the distance between the knob fixture center and cover block center, and σ is the standard deviation of both the knob fixture center and cover block center, which is 0.1 mm in our experiment. In addition, the magnetic field distortion is routinely corrected daily by the MRI scanner operator.

As for the manual localization results, we took the mean result from three experts to reduce random error. An influential factor for manual localization method is the magnetic susceptibility difference between brain tissue and the solution. The difference of the artifact shape lies in their difference in magnetic susceptibility. The magnetic susceptibility of Pt–Ir alloy is 231 ppm (C. Q. Jiang et al., 2013), while the brain tissue has a magnetic susceptibility in the range between –8.8 and –9.2 ppm, and our solution of –9 ppm (Duyn and Schenck, 2017). The influence of magnetic susceptibility to magnetic field distortion can be described as a distortion factor (Ladd et al., 1996): $\frac{\mu_{i}-\mu_{e}}{\mu_{i}+\mu_{e}},$ where μ_*i*_ and μ_*e*_ stands for the magnetic susceptibility of the material inside and outside the lead. In the extreme case of brain magnetic susceptibility of –9.2 and –8.8 ppm, the distortion factor is 1.1077 and 1.1028, respectively, which has a difference of 0.2% in contrast to 1.1052 for water. We consider the difference of 0.2% to be of minor influence.

There are differences in noise level between phantom and *in vivo* condition. The noise in clinical condition is higher because of the complex background condition. Here we use the blank area, which contains no medium that generates signal to estimate the noise level in both the phantom and *in vivo* data. The localization error was increased after we added Gaussian noise. A possible explanation is that the artifact outlines have been changed after adding artificial noise. These images after treatment will bring more difficulty for us to distinguish the center of artifact hypointensity area, which deteriorates the localization accuracy. However, the localization accuracy is still acceptable after our correction method. After correction, localization error is no larger than 1.6 mm. The mean error increased from 0.42 to 0.79 mm, which does not change the conclusion that MR can be used as a localization approach.

This study also analyzed the difference between the individual template and mean artifact template, and showed the advantages in using the individual template. It was revealed that the individual artifact template can have better registration result than the mean template.

Previous studies generally treated the geometric center of the hypointensive artifact in MR images as the contact center (Pollo et al., 2004; Thani et al., 2011), which was only valid under special scenarios, namely, with the lead orientation parallel to the magnetic field, and with the reconstruction direction parallel or perpendicular to the lead axis. In fact, the artifact appearance was strongly dependent on lead orientation. On the other hand, black artifacts are mainly magnetic sensitive artifacts, as well as RF artifacts, which will not only cause local blackening, but also cause some areas to brighten, which makes the situation more complicated. Generally speaking, the geometric center of the artifact tends to deviate further from the electrode center as the angle increases. Even in parallel positions, there is a small difference. This error can be reduced by simple empirical formula fitting. The more accurate analysis can be used to model and calculate the artifacts and make more detailed correction. On the other hand, the hypointensity area mainly consists of the susceptibility artifact, while RF artifacts will not only cause local hypo-intensity but also cause local hyperintensity, which will make the situation more complicated. Generally speaking, as the main field angle increases, the geometric center of the artifact tends to deviate further from the contact center. Even in parallel positions, there is a small deviation. This error can be reduced by a simple regression formula. More accurate analysis can be achieved by modeling and calculating the artifacts.

The traditional method uses averaged lead template to register subject data. However, after averaging, the template loses some detailed information of the contact artifact feature. The averaged template is not as good as the closest direction template, but still have the potential to achieve an acceptable result. There are differences between individual templates. Their paired correlation coefficient matrixes show that the template pair with a smaller direction difference has a higher similarity score. This result indicates that choosing a template that has a small direction difference with the patient lead will have a better localization result. On the other hand, the mean template has a stable performance in registration.

The CT/MRI fusion method is widely used because of the low tissue resolution of MRI. However, this approach assumes that the brain remains the same before and after the surgery, while previous studies show that the brain keeps changing after the surgery (Tessitore et al., 2017; Gao, 2018). Specifically, patients with Parkinson’s disease have cortical atrophy, and long-term DBS stimulation can lead to ventricular volume changes (Lewis et al., 2009), which will affect the accuracy of preoperative and postoperative registration. In addition, cerebrospinal fluid outflow can lead to brain drift (Elias et al., 2007; Matias et al., 2018), even up to 6.5 mm (Hamed et al., 2020). In addition, the accuracy of the CT/MR fusion has been changing for many years (Li et al., 2017). From 2000 to 2015, some operations were converted to frameless positioning, which improved the accuracy to a certain extent. However, more than 50% of the operations with frame positioning were still in use and were widely used, with the maximum deviation of 6 mm. Even the newly developed Leksell frameless system may achieve a positioning deviation of 2 mm (Li et al., 2017). The results of this paper, on the one hand, has an accuracy of 0.42 mm, and it is not necessary to conduct CT/MR fusion electrode positioning, and also can intuitively obtain the relative position relationship between the electrode and the nucleus from the postoperative data. Even if the electrode displacement or brain structure changes after operation, we will not be affected by the use of preoperative data. On the other hand, the traditional average template registration process is improved, and more accurate results are obtained with the individual template.

At present, for the relationship between the electrode implantation position and the activation area and even the curative effect, some studies have shown that the best implantation position for STN DBS in the treatment of Parkinson’s disease motor symptoms is the dorsolateral STN (Hamani et al., 2004; Herzog et al., 2004); a deviation of 2 mm may lead to 60% difference in clinical effect (Horn et al., 2019). In order to compensate for the deviation from the target, it is necessary to increase the stimulation intensity, which leads to excessive stimulation in the surrounding area, whose volume can reach one to six times the volume of the STN nucleus (Kramme et al., 2020). Other studies have shown that there are complex functional connections in the basal ganglia (Greene et al., 2020). Small deviations can lead to stimulation in other areas of the cortex. There are similar differences within STN, which need to be treated with caution (Haynes and Haber, 2013). The accuracy of electrode position judgment will affect the mechanism analysis and curative effect prediction. Therefore, Andy Horn et al. (2019) proposed a probability method to infer sweet spot. A previous study has shown that the fiber bundles with different angles from the electrodes have different thresholds under fixed stimulation mode (Slopsema et al., 2019), and false stimulation of the nucleus can affect up to 60% of the postoperative outcomes. Both IC and HDP pathways are located near STN, which are related to the auditory system and motor symptoms, respectively, and are parallel to, and perpendicular to, the electrode direction.

MRI is more and more widely used, and has gradually become a physical examination tool. Through repeated scanning, it can track the changes in the brain for a long time and master the evolution of the disease (Elias et al., 2007; Matias et al., 2018; Hamed et al., 2020). For DBS-implanted patients, repeated scanning of radiation CT is not appropriate. In the past, due to safety considerations, repeated MRI scans were not acceptable. However, with the increasing understanding of DBS MRI safety, as well as new compatible devices (C. Jiang et al., 2014; Mo et al., 2016), this restriction is being removed. MRI study of long-term changes after DBS implantation will become a normal. This provides an opportunity to continuously track brain changes under long-term DBS stimulation.

There is agreement between our results and previous research in which the center of the artifact coincides with the contact center in special cases when the lead axis is parallel to the main field. The fiducial system achieved a relatively accurate localization result due to the small artifact induced by the PMMA material. Thus, the contact coordinate calculated by our fiducial system can be considered as the real position.

Our method implies that error can be corrected by reducing deviation from the artifact center. Notice that in a practical situation, surgeons need to image two leads at the same time, and neither the MF angles will reach 0 degrees. Then our correction formula can be used.

## Conclusion

This study conducted the first assessment of MR-based lead localization deviation by measuring the deviation in coronal/transverse views, T1/T2 sequences, and different lead orientations and resliced directions. We also showed the advantage of the individual template compared with the mean template. These results indicate that the resliced direction and lead orientation are factors affecting the localization accuracy. More accurate localization results can be secured using carefully selected lead orientations and resliced directions, while taking the direction of the template into consideration when choosing a registration template.

## Data Availability Statement

The original contributions presented in the study are included in the article/supplementary material, further inquiries can be directed to the corresponding author/s.

## Author Contributions

CH conducted the study and prepared the manuscript with directions from CJ and LML. FZ provided important information about the experiment technique. LZL, CJ, and LML reviewed the manuscript. All the authors approved the manuscript.

## Conflict of Interest

The authors declare that the research was conducted in the absence of any commercial or financial relationships that could be construed as a potential conflict of interest.

## Publisher’s Note

All claims expressed in this article are solely those of the authors and do not necessarily represent those of their affiliated organizations, or those of the publisher, the editors and the reviewers. Any product that may be evaluated in this article, or claim that may be made by its manufacturer, is not guaranteed or endorsed by the publisher.
